# GABA, Selank, and Olanzapine Affect the Expression of Genes Involved in GABAergic Neurotransmission in IMR-32 Cells

**DOI:** 10.3389/fphar.2017.00089

**Published:** 2017-02-28

**Authors:** Elena Filatova, Anastasiya Kasian, Timur Kolomin, Ekaterina Rybalkina, Anelya Alieva, Lyudmila Andreeva, Svetlana Limborska, Nikolay Myasoedov, Galina Pavlova, Petr Slominsky, Maria Shadrina

**Affiliations:** ^1^Department of Molecular Basis of Human Genetics, Institute of Molecular Genetics, Russian Academy of SciencesMoscow, Russia; ^2^Laboratory of Tumor Cells Genetics, Blokhin Russian Cancer Research Center, Ministry of Health of the Russian FederationMoscow, Russia; ^3^Department of Chemistry of Physiologically Active Compounds, Institute of Molecular Genetics, Russian Academy of SciencesMoscow, Russia; ^4^Group of Neurogenetics and Developmental Genetics, Institute of Gene Biology, Russian Academy of SciencesMoscow, Russia

**Keywords:** Selank, GABA, olanzapine, IMR-32 cells, gene expression

## Abstract

Clinical studies have shown that Selank had an anxiolytic effect comparable to that of classical benzodiazepine drugs, which can enhance the inhibitory effect of GABA by allosteric modulation of GABA_A_ receptors. These data suggest that the molecular mechanism of the effect of Selank may also be related to its ability to affect the performance of the GABAergic system. To test this hypothesis, we studied the changes in expression of 84 genes involved in the functioning of the GABAergic system and in the processes of neurotransmission in the culture of neuroblastoma IMR-32 cells using qPCR method. As test substances, in addition to Selank, we selected the major GABA_A_ receptor ligand, GABA, the atypical antipsychotic, olanzapine, and combinations of these compounds (Selank and GABA; Selank and olanzapine). We found no changes in the mRNA levels of the genes studied under the effect of Selank. The combined effect of GABA and Selank led to nearly complete suppression of changes in expression of genes in which mRNA levels changed under the effect of GABA. When Selank was used in conjunction with olanzapine, the expression alterations of more genes were observed compared with olanzapine alone. The data obtained indicate that Selank has no direct effect on the mRNA levels of the GABAergic system genes in neuroblastoma IMR-32 cells. At the same time, our results partially confirm the hypothesis that the peptide may affect the interaction of GABA with GABA_A_ receptors. Our data also suggest that Selank may enhance the effect of olanzapine on the expression of the genes studied.

## Introduction

Drugs that are based on natural regulatory peptides are currently becoming more widely used. Synthetic analogs of regulatory peptides typically contain only natural amino acids in their structure, so that they practically do not have any toxic side effects. Drugs that are developed on the basis of regulatory peptides can provide directional effects on certain human body systems and are already used for the treatment of a variety of human diseases, such as cardiovascular disease (Gusev et al., 1997), gastrointestinal disease (Ivanov Iu and Iasnetsov, 2000), viral infections (Ershov et al., 2009; Andreeva et al., 2010), and various pathologies of the nervous system (Gusev et al., 2005).

Selank is a synthetic analog of the natural immunopeptide taftsin, belonging to a group of drugs of peptidic nature, and was developed at the Institute of Molecular Genetics of the Russian Academy of Sciences, in cooperation with the Zakusov Scientific Research Institute of Pharmacology. This peptide consists of the short fragment Thr-Lys-Pro-Arg of the heavy chain of the human immunoglobulin G and the tripeptide Pro-Gly-Pro at the end of the molecule, which provides metabolic stability and duration of action of the drug (Ashmarin et al., 2005; Ashmarin, 2007). Clinical trials of Selank have shown that this peptide can affect both the immune and the nervous system (Czabak-Garbacz et al., 2006; Semenova et al., 2008). It was shown that Selank had a pronounced anxiolytic effect comparable to that of classical benzodiazepine drugs (Seredenin et al., 1990, 1998). It is known that the classical benzodiazepines act via gamma-aminobutyric acid (GABA) type A receptors. They enhance the GABA effect by allosteric modulation, which increases the frequency of opening of channels for chlorine ions. The Selank action mechanism may be related to its ability to affect the performance of the GABAergic system.

Previously, it was shown that Selank causes a marked change in the expression of genes involved in inflammatory processes in the hippocampus and spleen of rodents (Kolomin et al., 2010, 2011, 2014). Our results have confirmed at the molecular level that the clinical effects observed after the introduction of Selank are related to its antiviral activity (Ershov et al., 2009; Andreeva et al., 2010).

Recent studies have shown that amount of specifically bound ligand ([^3^H]GABA) changes in the presence of Selank, and Selank preliminary intranasal administration causes a change in the number of GABA-specific binding sites but does not affect receptor affinity (V'yunova et al., 2014). Based on these data, the authors suggested that Selank can lead to a rapid change in the GABAergic system state by binding to GABA receptors and allosterically modulating the activity of GABA_A_ receptor. It is possible that the transcriptome changes that we previously identified are implemented partially via modulation of activity of GABA_A_ receptors by Selank. Earlier, we also found a positive correlation between the changes in the expression of genes involved in neurotransmission in the frontal cortex of rats within 1 h after administration of Selank or GABA. Our results showed that Selank caused a number of alterations in the expression of genes involved in the functioning of the GABAergic system and in the processes of neurotransmission (Volkova et al., 2016).

To test the hypothesis of a possible effect of Selank through the regulation of the activity of GABA_A_ receptors, we studied the changes in expression of 84 genes involved in neurotransmission in the IMR-32 cell line in response to Selank. The human neuroblastoma cell line, IMR-32, was chosen for study because these cells express functional GABA_A_ receptors (Anderson et al., 1993; Noble et al., 1993; Sapp and Yeh, 2000). To detect the effects associated with the action on the GABA_A_ receptor, we also conducted analysis of the changes in gene expression in response to GABA, a major GABA_A_ receptor ligand, and olanzapine, which is an atypical benzodiazepine that has the most pronounced affinity for 5-HT_2_ receptors (Bymaster et al., 1996).

## Materials and methods

### Cells and reagents

The human neuroblastoma cell line, IMR-32, was obtained from the A.T.C.C. (LGC Standards Sp. z.o.o., Poland). The cells were maintained in a humidified atmosphere containing 5% CO^2^ and 95% humidified air at 37°C in Dulbecco's modified Eagle's medium (DMEM) with L-glutamine (PanEco, Russia) supplemented with 10% fetal bovine serum (FBS) (PanEco), and gentamicin (50 mkg/ml) (Veropharm, Russia).

### Selank, GABA, and olanzapine treatment

IMR-32 cells were seeded into 6-well plates (Corning, The Netherlands) at 1–2 million cells per well in 4 ml of cell culture medium with Phenol Red per well, following incubation for 24 h at 37°C to allow the cells to adhere. After 24 h of incubation, physiological solution (50 mkl), Selank (1 nmol per well), GABA (1 nmol per well), olanzapine (1 nmol per well), a mixture of GABA and Selank (1 nmol of GABA and 1 nmol of Selank per well), or a mixture of Selank and olanzapine (1 nmol of Selank and 1 nmol of olanzapine per well) were added into the culture medium and the cells were incubated with the reagents for 1 h. The specified dose of Selank was selected as an optimum dose, that is used in studies of effects of peptides in cell cultures (Dolotov et al., 2015). All procedures were performed twice. After incubation with the reagents, the cells were washed with 1 ml of the physiological solution and immediately lyzed with 0.5 ml of Trizol reagent (Invitrogen, Thermo Fisher Scientific Inc.) per well. The lyzed cells were stored at −70°C prior to further procedures.

### RNA purification, reverse transcription, and quantitative real-time PCR (qPCR)

The lysates were incubated at −20°C for 1 h, then at +4°C for 1 h prior to RNA purification. Chloroform (0.1 ml) was added to each lysate. Tubes were shaken vigorously by hand for 15 s and incubated at room temperature for 3 min. After incubation, the samples were centrifuged at 12000 × g for 15 min at +4°C. The aqueous phase was placed into a new tube and the RNA isolation procedure was carried out using the QIAamp® RNA mini kit (Qiagen, Germany) according to the manufacturer's recommendations. RNA quality was monitored using an Experion automated electrophoresis system (Bio-Rad Laboratories). The RNA quality index was higher than 8.5 in all samples. First-strand cDNAs were synthesized using the RT^2^ First Strand Kit (Qiagen) according to the manufacturer's protocol. qPCR was performed using the Custom Human RT^2^ Profiler™ PCR Array: CAPH11633C (Qiagen). Amplification was carried out on the StepOnePlus™ Real-Time qPCR System (Life Technologies, USA) using the RT^2^ SYBR Green Mastermix (Qiagen, Germany). Thermal cycling was carried out as follows: (1) 95°C for 600 s, followed by (2) 40 cycles of 15 s at 95°C and 60 s at 60°C. All reactions were repeated three times for the cDNAs from each experimental and control cells. The qPCR study follows the MIQE guidelines.

### Statistical analysis

The threshold reaction cycle (*Cq*) values obtained for the genes studied were normalized to the *Cq*-values of the four reference genes: *TFRC, TSPO, B2M*, and *UBC*. Statistical data analysis of the normalized *Cq*-values and identification of significant differences between the levels of expression of the genes studied in nerve cells in the human neuroblastoma cell line IMR-32 after the incubation with the physiological solution and in the cells after the incubation with the substances studied was performed using the RT^2^ Profiler PCR Array Data Analysis version 3.5 (http://pcrdataanalysis.sabiosciences.com/pcr/arrayanalysis.php). Data analysis is based on the ΔΔC_q_ method with normalization of the raw data to reference genes. Genes in which the mRNA level changed significantly (*p* ≤ 0.05) 1.5 times or more were taken into account in the analysis of the changes in expression under the action of the test compounds. A comparison of significant changes of gene expression after the incubation with the substances studied was performed using the Spearman's rank correlation coefficient with Statistica v8.0 software. The gene set enrichment analysis (GSEA) of the genes studied and visualization of functional relations between proteins, encoded by these genes, were performed using Pathway Studio version 11.2.5.9 (Elsevier, USA).

## Results

We studied the effects of Selank, GABA, olanzapine, as well as combinations of these compounds (Selank and GABA; Selank and olanzapine) on the changes of mRNA levels of the 84 genes involved in neurotransmission processes in nerve cells in the human neuroblastoma cell line IMR-32. Preliminary analysis showed that the values of threshold cycles (*Cq*) of 15 genes studied (*BIRC3, CACNA1A, CX3CR1, DRD1, GABRA4, GABRA6, GABRD, GABRP, GABRR1, HCRT, IL2, MMP7, NPFFR2, SLC6A12*, and *PTGS2*) were higher than 35, which indicates the low representation of mRNA in cells examined. Therefore, these genes were excluded from further analysis.

The results of the expression analysis of the effects of Selank, GABA, olanzapine, and their combinations on the expression of 69 genes are shown in Table 1. After incubation of IMR-32 cells with GABA, 14 genes changed their expression with the majority of them (11) showing a decrease in the mRNA level. The mRNA levels of three genes increased: *GABRG2* increased 1.7 times, and *GABRA5* and *GNAQ* increased 1.6 times. Incubation of the cell culture with olanzapine resulted in changes of expression of 25 genes: transcript levels of 21 genes were decreased. The decrease in expression of three genes was especially pronounced in *CSF2* (4.5 times), *FOS* (3 times), and *JUNB* (5.3 times). Four genes (*GABRA5, GABRG2, GNACQ*, and *SNCA*) showed an increase of expression of no more than 2-fold compared with the control cells. It should be noted that there were no changes in the mRNA levels of the genes studied under the effect of Selank in the IMR-32 cells.

**Table 1:** **The relative mRNA levels of genes involved in neurotransmission processes in neuroblastoma IMR-32 cell culture after incubation with GABA, olanzapine, Selank, Selank with GABA, or Selank with olanzapine**.

**Gene Symbol**	**Official full name**	**GABA**		**Olanzapine**		**Selank**		**Selank + GABA**		**Selank + Olanzapine**	
		**Fold Regulation**	***p*-value**	**Fold Regulation**	***p*-value**	**Fold Regulation**	***p*-value**	**Fold Regulation**	***p*-value**	**Fold Regulation**	***p*-value**
*ABAT*	4-aminobutyrate aminotransferase	−1.43	0.042	**−1.89**	0.001	−1.01	0.941	−1.34	0.017	**−1.96**	0.002
*ADCY7*	Adenylate cyclase 7	−2.00	0.066	**−2.30**	0.039	1.00	0.934	−1.46	0.145	**−2.84**	0.023
*ADORA1*	Adenosine A1 receptor	−1.28	0.346	**−1.50**	0.047	−1.06	0.592	−1.20	0.241	**−1.77**	0.011
*ADORA2A*	Adenosine A2a receptor	**−3.82**	0.040	−2.94	0.072	−1.46	0.237	−2.71	0.059	**−3.88**	0.032
*ALDH5A1*	Aldehyde dehydrogenase 5 family, member A1	−1.20	0.251	**−1.51**	0.008	−1.02	0.761	−1.15	0.136	**−1.65**	0.005
*BCL2*	B-cell CLL/lymphoma 2	−1.04	0.670	−1.33	0.099	−1.04	0.701	−1.18	0.209	−1.30	0.116
*BCL2L1*	BCL2-like 1	−1.75	0.050	**−2.22**	0.005	−1.10	0.362	−1.25	0.096	**−2.62**	0.002
*BDNF*	Brain-derived neurotrophic factor	1.06	0.547	1.06	0.352	1.08	0.349	1.07	0.440	1.15	0.126
*BIRC2*	Baculoviral IAP repeat containing 2	1.35	0.039	1.42	0.011	1.02	0.779	1.16	0.138	**1.57**	0.004
*CACNA1B*	Calcium channel, voltage-dependent, N type, alpha 1B subunit	−1.71	0.065	**−2.18**	0.041	−1.25	0.338	−1.56	0.103	**−2.75**	0.019
*CCND1*	Cyclin D1	−1.01	0.954	−1.17	0.067	−1.04	0.560	−1.02	0.651	−1.27	0.106
*CSF2*	Colony stimulating factor 2 (granulocyte-macrophage)	**−3.07**	0.005	**−4.48**	0.010	1.11	0.617	−1.43	0.392	**−6.21**	0.003
*CX3CL1*	Chemokine (C-X3-C motif) ligand 1	**−2.31**	0.004	**−2.70**	0.026	1.06	0.712	−1.29	0.286	**−3.74**	0.004
*DRD2*	Dopamine receptor D2	−1.46	0.102	**−1.65**	0.042	−1.05	0.650	−1.18	0.228	**−1.92**	0.011
*DRD3*	Dopamine receptor D3	**−1.56**	0.026	−2.35	0.125	1.23	0.301	−1.34	0.382	**−3.02**	0.030
*DRD4*	Dopamine receptor D4	−1.70	0.115	−1.69	0.139	−1.19	0.489	−1.30	0.323	−2.15	0.056
*DRD5*	Dopamine receptor D5	−1.07	0.723	−1.44	0.457	1.25	0.705	1.18	0.783	−1.81	0.427
*EGR1*	Early growth response 1	−1.28	0.240	−1.70	0.112	−1.14	0.593	−1.17	0.517	−1.89	0.059
*FN1*	Fibronectin 1	**−1.59**	0.009	**−2.06**	0.001	1.01	0.792	−1.28	0.029	**−2.23**	0.0003
*FOS*	FBJ murine osteosarcoma viral oncogene homolog	−1.76	0.084	**−3.06**	0.001	−1.21	0.293	−1.29	0.061	**−3.63**	0.002
*GABBR1*	Gamma-aminobutyric acid (GABA) B receptor, 1	−2.11	0.121	−2.38	0.097	−1.01	0.820	−1.34	0.320	**−3.11**	0.044
*GABBR2*	Gamma-aminobutyric acid (GABA) B receptor, 2	−1.13	0.534	−1.11	0.620	1.07	0.771	1.06	0.745	−1.25	0.357
*GABRA1*	Gamma-aminobutyric acid (GABA) A receptor, alpha 1	−1.36	0.106	**−1.54**	0.021	−1.14	0.318	−1.13	0.340	−1.35	0.086
*GABRA2*	Gamma-aminobutyric acid (GABA) A receptor, alpha 2	1.33	0.076	1.35	0.022	−1.06	0.477	1.24	0.039	1.33	0.039
*GABRA3*	Gamma-aminobutyric acid (GABA) A receptor, alpha 3	1.16	0.083	1.09	0.436	1.03	0.782	1.11	0.243	−1.00	0.981
*GABRA5*	Gamma-aminobutyric acid (GABA) A receptor, alpha 5	**1.60**	0.026	**1.62**	0.009	1.05	0.641	1.25	0.059	**1.86**	0.005
*GABRB1*	Gamma-aminobutyric acid (GABA) A receptor, beta 1	−1.28	0.485	−1.56	0.217	1.12	0.759	−1.00	0.932	−1.58	0.303
*GABRB2*	Gamma-aminobutyric acid (GABA) A receptor, beta 2	1.32	0.082	1.24	0.148	−1.05	0.945	1.11	0.595	1.29	0.104
*GABRB3*	Gamma-aminobutyric acid (GABA) A receptor, beta 3	1.32	0.001	1.09	0.060	1.04	0.191	1.07	0.011	1.12	0.002
*GABRE*	Gamma-aminobutyric acid (GABA) A receptor, epsilon	−1.63	0.185	−1.53	0.354	1.08	0.800	1.10	0.889	−1.79	0.137
*GABRG1*	Gamma-aminobutyric acid (GABA) A receptor, gamma 1	1.37	0.069	1.38	0.065	1.01	0.767	1.10	0.541	1.24	0.270
*GABRG2*	Gamma-aminobutyric acid (GABA) A receptor, gamma 2	**1.65**	0.018	**1.74**	0.012	1.38	0.216	1.47	0.045	**1.74**	0.007
*GABRG3*	Gamma-aminobutyric acid (GABA) A receptor, gamma 3	1.19	0.149	1.03	0.838	1.32	0.227	1.09	0.476	−1.05	0.608
*GABRQ*	Gamma-aminobutyric acid (GABA) receptor, theta	**−1.62**	0.037	−1.92	0.105	−1.08	0.613	−1.45	0.194	**−2.62**	0.037
*GABRR2*	Gamma-aminobutyric acid (GABA) receptor, rho 2	−2.03	0.063	**−2.47**	0.043	1.01	0.905	−1.50	0.204	**−2.78**	0.037
*GAD1*	Glutamate decarboxylase 1 (brain, 67kDa)	−1.07	0.622	−1.36	0.468	1.24	0.785	1.15	0.860	−2.06	0.249
*GLS*	Glutaminase	1.20	0.043	1.16	0.039	1.01	0.857	1.11	0.019	1.24	0.003
*GLUL*	Glutamate-ammonia ligase	−1.01	0.798	−1.14	0.001	1.02	0.564	1.02	0.594	−1.17	0.002
*GNAI1*	Guanine nucleotide binding protein (G protein), alpha inhibiting activity polypeptide 1	1.22	0.064	1.23	0.062	1.05	0.587	1.18	0.067	1.34	0.008
*GNAQ*	Guanine nucleotide binding protein (G protein), q polypeptide	**1.57**	0.039	**1.69**	0.014	1.07	0.504	1.24	0.055	**1.87**	0.001
*GPHN*	Gephyrin	1.41	0.006	1.25	0.022	1.11	0.198	1.29	0.008	1.34	0.005
*GPR85*	G protein-coupled receptor 85	−1.15	0.218	−1.43	0.015	−1.24	0.073	−1.15	0.056	**−1.58**	0.004
*HCRTR2*	Hypocretin (orexin) receptor 2	−1.02	0.826	−1.12	0.835	1.30	0.555	1.05	0.978	1.01	0.822
*HTR1B*	5-hydroxytryptamine (serotonin) receptor 1B	−2.32	0.153	−1.97	0.214	1.06	0.971	−1.57	0.296	−2.28	0.146
*HTR2A*	5-hydroxytryptamine (serotonin) receptor 2A	−1.59	0.054	**−2.25**	0.045	−1.38	0.681	−1.23	0.709	−2.55	0.051
*HTR3A*	5-hydroxytryptamine (serotonin) receptor 3A	**−1.90**	0.012	**−2.25**	0.007	−1.15	0.568	−1.49	0.073	**−2.83**	0.001
*HTR3B*	5-hydroxytryptamine (serotonin) receptor 3B	−1.06	0.771	−1.26	0.563	1.21	0.785	1.32	0.636	−1.48	0.551
*ICAM1*	Intercellular adhesion molecule 1	−1.45	0.139	−2.30	0.054	1.05	0.796	−1.11	0.892	**−2.67**	0.015
*JUN*	Jun proto-oncogene	−1.47	0.130	**−2.01**	0.003	−1.20	0.072	−1.34	0.035	**−2.37**	0.001
*JUNB*	Jun B proto-oncogene	**−3.14**	0.042	**−5.19**	0.002	−1.23	0.257	**−1.71**	0.034	**−6.57**	0.002
*MCHR1*	Melanin-concentrating hormone receptor 1	−1.27	0.148	**−1.58**	0.011	1.05	0.689	−1.37	0.004	**−1.84**	0.001
*MMP10*	Matrix metallopeptidase 10 (stromelysin 2)	**−2.25**	0.002	**−2.79**	0.018	−1.34	0.179	−1.53	0.328	**−3.50**	0.006
*MYC*	V-myc myelocytomatosis viral oncogene homolog (avian)	−1.21	0.104	−1.49	0.008	−1.08	0.563	−1.25	0.440	−1.17	0.235
*NOS2*	Nitric oxide synthase 2, inducible	−1.09	0.859	−1.36	0.160	1.15	0.468	1.08	0.724	−1.60	0.056
*NPFFR1*	Neuropeptide FF receptor 1	**−2.11**	0.044	−2.25	0.072	1.07	0.832	−1.35	0.357	**−3.79**	0.020
*NSF*	N-ethylmaleimide-sensitive factor	1.13	0.0004	−1.03	0.155	1.02	0.190	1.02	0.190	1.00	0.689
*ODC1*	Ornithine decarboxylase 1	1.34	0.027	1.34	0.027	1.16	0.083	1.22	0.023	**1.52**	0.007
*P2RX7*	Purinergic receptor P2X, ligand-gated ion channel, 7	1.41	0.550	1.11	0.972	1.54	0.405	1.49	0.451	−1.02	0.736
*PRKCA*	Protein kinase C, alpha	−1.29	0.015	**−1.57**	0.0005	−1.01	0.853	−1.17	0.068	**−1.70**	0.0003
*PRKCE*	Protein kinase C, epsilon	−1.12	0.446	−1.26	0.028	1.08	0.252	−1.03	0.766	**−1.51**	0.003
*PRLHR*	Prolactin releasing hormone receptor	−1.84	0.168	−2.18	0.114	1.23	0.584	−1.07	0.941	−2.73	0.102
*SLC1A3*	Solute carrier family 1 (glial high affinity glutamate transporter), member 3	−1.16	0.453	−1.30	0.300	1.09	0.809	−1.17	0.433	−1.48	0.161
*SLC32A1*	Solute carrier family 32 (GABA vesicular transporter), member 1	**−1.73**	0.050	−2.32	0.075	1.02	0.838	−1.33	0.416	**−4.01**	0.010
*SLC38A1*	Solute carrier family 38, member 1	1.20	0.087	1.19	0.014	1.00	0.966	1.05	0.253	1.24	0.013
*SLC6A1*	Solute carrier family 6 (neurotransmitter transporter, GABA), member 1	−1.42	0.302	−1.84	0.134	1.19	0.573	1.00	0.888	−2.30	0.114
*SLC6A11*	Solute carrier family 6 (neurotransmitter transporter, GABA), member 11	−1.67	0.072	**−1.88**	0.044	−1.07	0.674	−1.38	0.178	**−2.15**	0.031
*SLC6A13*	Solute carrier family 6 (neurotransmitter transporter, GABA), member 13	−1.15	0.561	−1.37	0.267	1.30	0.343	1.13	0.717	−1.56	0.143
*SLC8A3*	Solute carrier family 8 (sodium/calcium exchanger), member 3	−1.20	0.379	−1.52	0.095	−1.10	0.514	−1.11	0.514	**−1.83**	0.039
*SNCA*	Synuclein, alpha (non A4 component of amyloid precursor)	1.47	0.113	**1.71**	0.026	−1.09	0.624	1.17	0.358	**1.84**	0.013

*The data in bold denote changes in the expression by 1.5 times or more, with a significance level (p < 0.05). The red color denotes the increase in expression of genes; the blue color denotes the decrease in expression of genes*.

Statistically significant change in the expression of only one gene, *JUNB*, whose mRNA level decreased 1.7 times, was shown after incubation of the IMR-32 cells simultaneously with Selank and GABA. At the same time, changes in the mRNA levels of the greatest number of genes were shown after incubation of the IMR-32 cells simultaneously with Selank and olanzapine when compared with all compounds and their combinations studied. Significant changes in the expression of 35 of 69 genes chosen for the analysis were shown: 29 genes showed decreased mRNA levels, and transcript levels of 10 of them (*ADORA2A, CSF2, CX3CL1, DRD3, FOS, GABBR1, JUNB, MMP10, NPFFR1*, and *SLC32A1*) decreased more than 3 times. The mRNA levels of six genes (*BIRC2, GABRA5, GABRG2, GNAQ, ODC1*, and *SNCA*) increased after the incubation of the IMR-32 cells with Selank and olanzapine.

The cumulative analysis of all obtained statistically significant data showed that after the incubation of IMR-32 neuroblastoma cells with all the compounds and their combinations a decrease in mRNA levels of most of the genes studied (31) was observed; in contrast, mRNA levels of only six genes (*BIRC2, GABRA5, GABRG2, GNAQ, ODC1*, and *SNCA*) were increased.

It should also be noted that the most pronounced change in expression was observed for two genes, *CSF2* and *JUNB*, after incubation of cells with the compounds studied. mRNA levels of these genes decreased three times after incubation with GABA, 4.5 and 5.3 times after incubation with olanzapine, and 6.3 and 6.7 times after incubation with Selank and olanzapine. Moreover, the *JUNB* gene was characterized by a significant 1.7-fold decrease in expression after incubation of the cells with GABA and Selank. This gene is the only one in which mRNA levels changed significantly in the four variants of incubation of IMR-32 cells with various compounds (namely, GABA; olanzapine; Selank and GABA; Selank and olanzapine).

**Figure 1:**
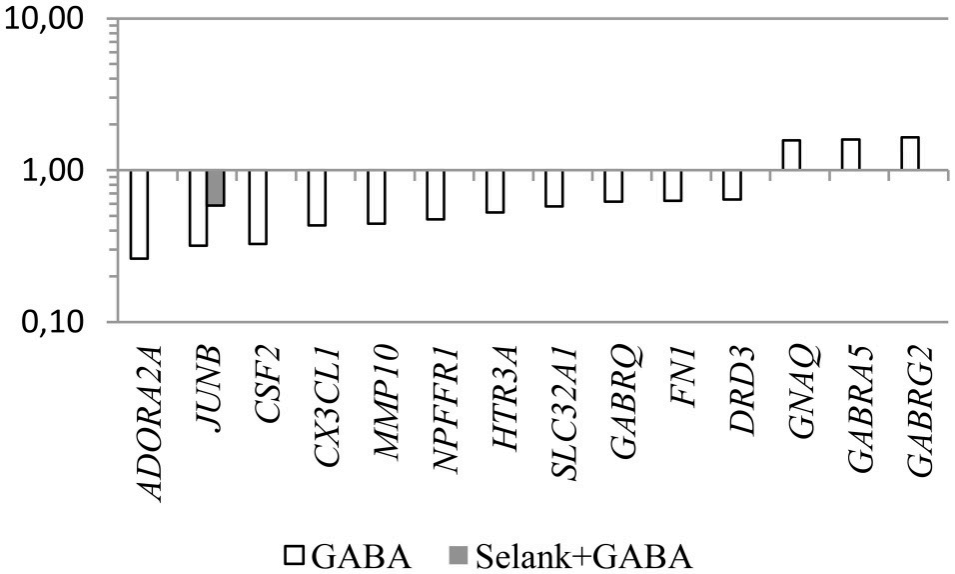
**The relative change in gene expression in neuroblastoma cells IMR-32 after incubation with GABA and after incubation with Selank and GABA**. The figure shows only statistically significant results (*p* ≤ 0.05). mRNA levels of genes in the control group were taken as 1.

**Figure 2:**
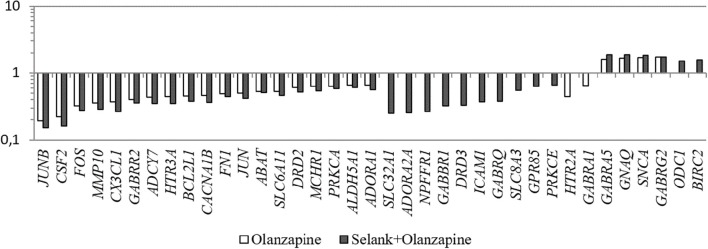
**The relative change in gene expression in neuroblastoma cells IMR-32 after incubation with olanzapine and after incubation with olanzapine and Selank**. The figure shows only statistically significant results (*p* ≤ 0.05). mRNA levels of genes in the control group were taken as 1.

Another interesting feature is that despite the fact that no statistically significant change in expression of the genes under study was observed under the effect of Selank, the combined effect of GABA and Selank led to nearly complete suppression of changes in expression of genes in which mRNA levels changed under the effect of GABA (Figure 1). When Selank was used in conjunction with olanzapine, the expression alterations of more genes were observed compared with olanzapine alone. Furthermore, changes in mRNA levels became more pronounced (Figure 2).

The correlation analysis was conducted for genes in which the mRNA levels significantly changed under the effect of the compounds studied. As a result, we discovered a very strong positive correlation between gene expression changes under the effect of GABA and those under the effect of olanzapine (*r* = 0.98, *p* ≤ 0.05).

We also conducted the Gene Set Enrichment Analysis (GSEA) of the genes which mRNA levels changed significantly in neuroblastoma cells IMR-32 after incubation with the mixture of Selank and olanzapine. The results are shown in Table 2. This group of genes was chosen for the analysis because the mixture of Selank and olanzapine caused changes in expression of the largest number of the genes when compared with other substances studied. Moreover, almost all of the genes, which expression was significantly affected by olanzapine, were also in the group that was chosen for GSEA.

**Table 2:** **Biological processes that were revealed by GSEA of the genes which mRNA levels changed significantly in neuroblastoma cells IMR-32 after incubation with the mixture of Selank and olanzapine**.

**Name of biological process**	**GO ID**	**Number of entities**	**Overlap**	**Overlapping entities**	***p*-value**
Gamma-aminobutyric acid signaling pathway	7214	29	7	*GABRR2; GABRA2; GABBR1; GABRA5; GABRB3; GABRG2; GABRQ*	1.15E-13
Transmembrane transport	55085	805	13	*SLC8A3; GABRB3; ADCY7; GABRA5; CACNA1B; SLC6A11; SLC32A1; SLC38A1; GABRR2; GABRQ; GABRA2; HTR3A; GABRG2*	3.22E-09
Neurotransmitter secretion	7269	72	6	*ALDH5A1; CACNA1B; SLC6A11; SLC32A1; ABAT; GLS*	6.27E-09
Ion transport	6811	627	11	*GABRA5; CACNA1B; SLC32A1; SLC38A1; GABRR2; GABRQ; SLC8A3; GABRA2; GABRB3; HTR3A; GABRG2*	2.86E-08
Signal transduction	0007165; 0023033	1843	17	*ADORA1; ADCY7; GNAQ; GABRB3; GNAI1; DRD3; DRD2; GABRA5; GABRR2; GABRQ; MCHR1; GPR85; PRKCE; ADORA2A; GABBR1; PRKCA; GABRG2*	3.08E-08
Response to organic cyclic compound	14070	253	8	*JUNB; BIRC2; ICAM1; BCL2L1; CACNA1B; FOS; JUN; PRKCA*	3.31E-08
Response to drug	0042493; 0017035	509	10	*DRD3; DRD2; JUNB; ICAM1; SLC6A11; FOS; JUN; ABAT; SNCA; ADORA2A*	4.73E-08
Ion transmembrane transport	34220	291	7	*GABRA2; GABRA5; GABRB3; GABRR2; GABRQ; HTR3A; GABRG2*	1.63E-06

## Discussion

Clinical studies have shown that Selank is highly effective in the prevention and treatment of generalized anxiety disorder and neurasthenia, as well as stress and anxiety. This effect of the peptide is similar to that of classical benzodiazepine drugs (Seredenin et al., 1990, 1998), which can enhance the inhibitory effect of GABA by allosteric modulation of GABA_A_ receptors. This suggests that the molecular mechanism of the effect of Selank may also be related to its ability to affect the GABA receptors.

We assessed changes in the expression of 84 genes involved in the functioning of the GABAergic system and in the processes of neurotransmission in the culture of neuroblastoma IMR-32 cells. It was shown that this cell line expresses predominantly functional GABA_A_ receptors (Anderson et al., 1993; Noble et al., 1993; Sapp and Yeh, 2000), and therefore was chosen for our study. As test substances, in addition to Selank, we selected the primary ligand of the GABA_A_ receptor, GABA, and the atypical antipsychotic, olanzapine, which has an affinity for the serotonin 5-HT_2_-receptor.

The absence of any changes in the mRNA levels of genes studied after the incubation of the cells with Selank suggests that Selank is not able to directly affect the activity of the GABAergic system in IMR-32 cell culture. We previously demonstrated that Selank changes the expression of significant amounts of genes involved in neurotransmission processes in neuronal cells in the frontal cortex of rats (Volkova et al., 2016). Similar changes in the expression of these genes were also observed upon administration of GABA. The data obtained earlier indicate that Selank is able to allosterically modulate the work of the GABAergic system. It is also known that the interaction of some allosteric modulators with the GABA receptor is determined by the subunit composition of this receptor (Sieghart, 1995; Zezula et al., 1996; Rudolph and Knoflach, 2011). Currently, the subunit composition of GABA_A_ receptors present in neuroblastoma IMR-32 cell culture is not precisely defined. mRNAs of some genes that code α1, α3, α4, β1, β3, γ2, and δ subunits of GABA_*A*_ receptors were discovered in this cell culture (Sapp and Yeh, 2000). However, presently, only the expression of α3, β1, β3, and γ2 subunits has been confirmed at the protein level by Western blot hybridization and electrophysiological methods (Noble et al., 1993; Sapp and Yeh, 2000). These data indicate that the variants of GABA_A_-receptors, the subunit composition of which is very limited, function in the IMR-32 cell line. Thus, it can be assumed that Selank has no direct effect on GABA_A_ receptors, presented in the IMR-32 cell culture, due to the composition of the receptor subunits included in these receptors.

Despite the fact that the effect of Selank in the cell culture investigated appears to be mediated by mechanisms unrelated to a direct interaction with the GABA_A_ receptor, the peptide is able to change the affinity of the GABA to the GABA_A_ receptor (V'yunova et al., 2014). Previously, it was shown that Selank is able to affect the specific binding of GABA to GABA_A_ receptors that may be caused by modulating properties of the peptide, which appear to consist of a change of the affinity of the endogenous ligands for the receptor under the effects of Selank on the receptor (V'yunova et al., 2014). We can assume that the reduction in the number of genes that changed their expression from 14 (cell culture when incubated with GABA) to one gene (incubation with Selank and GABA) partially support the hypothesis of a possible effect of the peptide through the regulation of GABAergic system activity.

It should be noted that, although olanzapine is an atypical neuroleptic with pronounced affinity and activity for the serotonin 5-HT_2_ receptor (Bymaster et al., 1996), the mechanism of action of olanzapine may also be associated with the effect on the GABAergic system. Thus, Skilbeck et al. have shown that atypical antipsychotics, such as olanzapine, affect the density of GABA_A_ receptors in the prefrontal cortex (Skilbeck et al., 2007, 2008). Furthermore, the anxiolytic effect of olanzapine (Moore et al., 1992; Inoue et al., 1996; Fu et al., 2000; Nemeroff, 2005) may be associated with increasing concentrations of allopregnanolone (Marx et al., 2000, 2003), which increases the frequency and duration of opening of channels for chlorine ions and enhances the inhibitory effect of GABA by binding to GABA_A_ receptors (Paul and Purdy, 1992; Twyman and MacDonald, 1992). Our data confirm that olanzapine is able to affect the expression of the GABAergic system genes, providing a pronounced effect on the mRNA levels of genes studied in the culture of the neuroblastoma IMR-32 cells. The presence of a pronounced positive correlation between changes in gene expression under the effect of GABA and olanzapine also provides support. It was demonstrated that olanzapine has a very weak affinity for the GABA_A_ receptor, which is represented in various tissues of humans and rats (Bymaster et al., 1996). It can be assumed that the features of the subunit composition of GABA_A_ receptors of this cell type affects the receptor affinity to olanzapine. On the other hand, the observed changes in the mRNA levels of the genes studied after the incubation of IMR-32 cell culture with olanzapine may be mediated by its effects on receptor systems that differ from the GABAergic system. Although neuroblastoma cell line IMR-32 predominantly express functional GABA_A_ receptors, the receptors of other systems (for example, nicotinic and muscarinic acetylcholine receptors) are present in the cell culture (Fraser and Lee, 1995; Gopalakrishnan et al., 2011).

Moreover, the data obtained indicate that the number of genes that changed their expression after the incubation with mixture of Selank and olanzapine is higher than the number of genes whose mRNA levels changed after the incubation with olanzapine alone. In addition, changes of transcript levels became more pronounced, which indicates that Selank may enhance the effect of olanzapine on expression of the genes studied. Unfortunately, although we currently do not have a detailed explanation for this phenomenon, we can make the following supposition: olanzapine binds to the GABA_A_ receptor and changes its structure so that Selank becomes capable of allosterically modulating the receptor and thereby enhances the effect of olanzapine in the cell culture studied.

**Figure 3:**
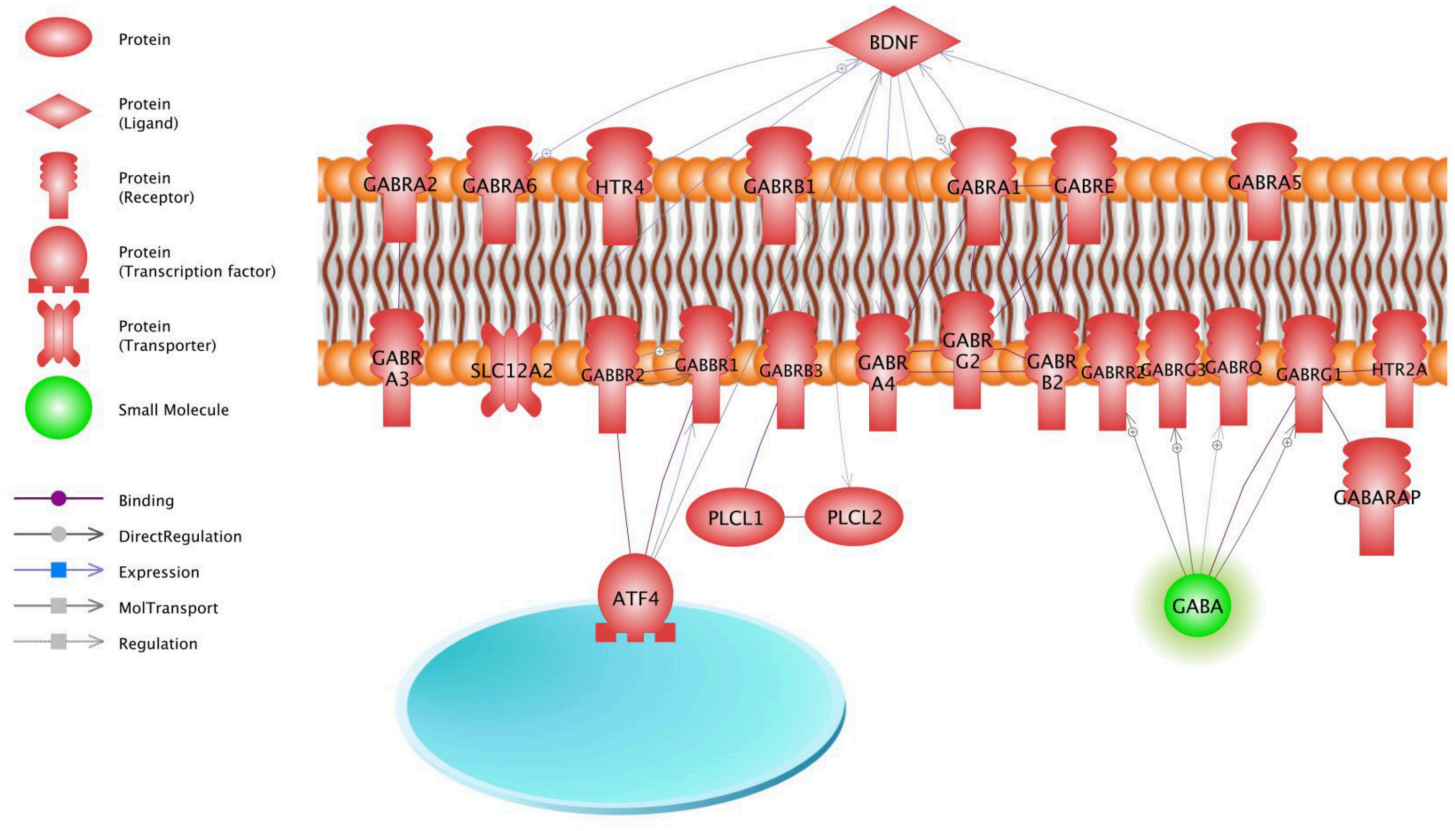
**Gamma-aminobutyric acid signaling pathway**.

The GSEA of the genes which mRNA levels changed significantly in neuroblastoma cells IMR-32 after incubation with the mixture of Selank and olanzapine showed that proteins encoded by these genes play important roles in processes of signal transduction in neurons, because biological processes, revealed by Gene Ontology enrichment analysis, are involved in neurotransmission. We also analyzed “gamma-aminobutyric acid signaling pathway,” the most significant of the biological processes identified, and built a scheme that visualized a functional relations between entities of this biological process and where brain derived neurotrophic factor (BDNF) played central role (Figure 3). It was shown that Selank may regulate expression of *Bdnf* in rat hippocampus after intranasal administration (Inozemtseva et al., 2008). Therefore, the observed changes in expression of the genes studied in neuroblastoma cells IMR-32 after incubation with the mixture of Selank and olanzapine suggest that Selank may modulate the action of olanzapine by affecting BDNF.

Thus, the data obtained indicate that Selank has no direct effect on the expression of genes of the GABAergic system in neuroblastoma IMR-32 cells. We can assume, that difference between expression profiles after the incubation with GABA and mixture of Selank and GABA partially confirms the hypothesis that the peptide may affect the interaction of GABA with GABA_A_ receptors.

## Author contributions

EF, TK, ER, GP, and LA performed the experimental work. AK, AA, MS, and PS undertook all statistical analyses and helped with their interpretation. PS and MS designed the study. AK and MS wrote the first draft of the manuscript. MS and PS contributed to the final writing of the manuscript. SL and NM was involved in revising the manuscript critically for important intellectual content. All authors contributed to and have approved the final manuscript.

### Conflict of interest statement

The authors declare that the research was conducted in the absence of any commercial or financial relationships that could be construed as a potential conflict of interest.
